# Effect of computed tomography value error on dose calculation in adaptive radiotherapy with Elekta X‐ray volume imaging cone beam computed tomography

**DOI:** 10.1002/acm2.13384

**Published:** 2021-08-10

**Authors:** Takuya Taniguchi, Takanori Hara, Tomohiro Shimozato, Fuminori Hyodo, Kose Ono, Shuto Nakaya, Yoshifumi Noda, Hiroki Kato, Osamu Tanaka, Masayuki Matsuo

**Affiliations:** ^1^ Department of Radiation Oncology Asahi University Hospital Gifu Japan; ^2^ Department of Radiology Gifu University Gifu Japan; ^3^ Department of Medical Technology Nakatsugawa Municipal General Hospital Gifu Japan; ^4^ Faculty of Radiological Technology School of Health Sciences Gifu University of Medical Science Seki Japan; ^5^ Department of Radiology Frontier Science for Imaging School of Medicine Gifu University Gifu Japan

**Keywords:** adaptive radiotherapy, cone beam computed tomography (CBCT), CT value

## Abstract

**Purpose:**

We evaluated the effect of changing the scan mode of the Elekta X‐ray volume imaging cone beam computed tomography (CBCT) on the accuracy of dose calculation, which may be affected by computed tomography (CT) value errors in three dimensions.

**Methods:**

We used the electron density phantom and measured the CT values in three dimensions. CT values were compared with planning computed tomography (pCT) values for various materials. The evaluated scan modes were for head and neck (S‐scan), chest (M‐scan), and pelvis (L‐scan) with various collimators and filter systems. To evaluate the effects of the CT value error of the CBCT on dose error, Monte Carlo calculations of dosimetry were performed using pCT and CBCT images.

**Results:**

The L‐scan had a CT value error of approximately 800 HU at the isocenter compared with the pCT. Furthermore, inhomogeneity in the longitudinal CT value profile was observed in the bone material. The dose error for ±100 HU difference in CT values for the S‐scan and M‐scan was within ±2%. The center of the L‐scan had a CT error of approximately 800 HU and a dose error of approximately 6%. The dose error of the L‐scan occurred in the beam path in the case of both single field and two parallel opposed fields, and the maximum error occurred at the center of the phantom in the case of both the 4‐field box and single‐arc techniques.

**Conclusions:**

We demonstrated the three‐dimensional CT value characteristics of the CBCT by evaluating the CT value error obtained under various imaging conditions. It was found that the L‐scan is considerably affected by not having a unique bowtie filter, and the S‐scan without the bowtie filter causes CT value errors in the longitudinal direction. Moreover, the CBCT dose errors for the 4‐field box and single‐arc irradiation techniques converge to the isocenter.

## 1 INTRODUCTION

Image‐guided radiotherapy (IGRT), widely recognized for its usefulness, employs diagnostic imaging technology to detect treatment sites accurately and enable highly accurate irradiation.^1^, ^2^ In particular, IGRT using a kilovoltage cone beam computed tomography (CBCT) obtains three‐dimensional (3D) cross‐sectional images similar to a planning computed tomography (pCT) and has high matching accuracy even in locations where position matching is difficult with two‐dimensional images.^3^, ^4^ Furthermore, because a CBCT can be used to observe the shapes of tumors and organs as well as changes in their positional relationships over the course of treatment, adaptive radiotherapy (ART) can be used to optimize the treatment plan according to the state of treatment.^5^, ^6^ However, CBCTs are more affected by scattered radiation and the heel effect than is computed tomography (CT), making it difficult to obtain accurate 3D CT values.^7^, ^8^, ^9^, ^10^ Therefore, various methods have been developed to improve the accuracy of CT values and maintain image uniformity, such as deformable image registration‐based dose verification for CBCT images,^11^, ^12^ a dual‐energy CBCT to improve image quality and dose calculation accuracy,^13^, ^14^ deep neural networks to improve the image quality of CBCT images while preserving anatomical structures,^15^, ^16^ and scattered radiation correction.^17^ These technologies have not yet been added to common commercial linear accelerator (linac). Contemporary commercial linacs are increasingly providing CT value correction for a CBCT.^18^, ^19^, ^20^ However, CT value correction depends on the imaging conditions and patient size.^21^, ^22^, ^23^, ^24^ We need to recognize the characteristics of the CT error of the CBCT and the dose error of ART using a CBCT to perform clinical radiotherapy more safely. However, although a CBCT uses a cone beam, CT values are evaluated only in the axial plane,^25^, ^26^ and a detailed evaluation in the longitudinal direction is not performed for most cases. Similarly, the dose error of the CBCT treatment plan has not been evaluated considering the details of the 3D CT value error of the CBCT. Recently, 3D treatment planning has become increasingly commonplace for high‐precision treatment. However, there is no report on the evaluation of the 3D CT error of a CBCT in commercial linacs, which is a problem that needs to be addressed at the earliest. Therefore, we evaluated in detail the 3D CT value error of the CBCT mounted on a common commercial linac, as well as the effect of the CT value error on the dose error for 3D radiation therapy planning.

## 2 MATERIALS AND METHODS

### 2.1 Materials

The Synergy X‐ray volume imaging (XVI) system (Elekta Ltd, Crawley, UK) installed in the radiotherapy device comprises an X‐ray tube and flat‐panel detector and can acquire X‐ray photographs, X‐ray fluoroscopic images, and CBCT images. The CBCT device is mounted on a linac. In this study, CT values of a CBCT were previously corrected using Catphan (Model 504, Phantom Laboratory, Salem, NY, USA). CT value correction was obtained from the density of air‐ and water‐equivalent material at the isocenter, based on the pCT (Alexion, Toshiba Medical Systems, Tokyo, Japan), according to the optional Hounsfield unit (HU) calibration procedure available for the Synergy XVI system. The CT value of a CBCT is mainly affected by the collimator, filter, and X‐ray voltage; therefore, HU calibration of the CBCT was performed by scanning the Catphan under all imaging conditions, and the CT values are corrected for each imaging condition. The CT values were measured using the 3‐slice averaged image of the Catphan Sensitometry Module. The CT values registered in XVI were averaged over the voxel size of 3.5 mm of the water and air inserts.

**FIGURE 1:**
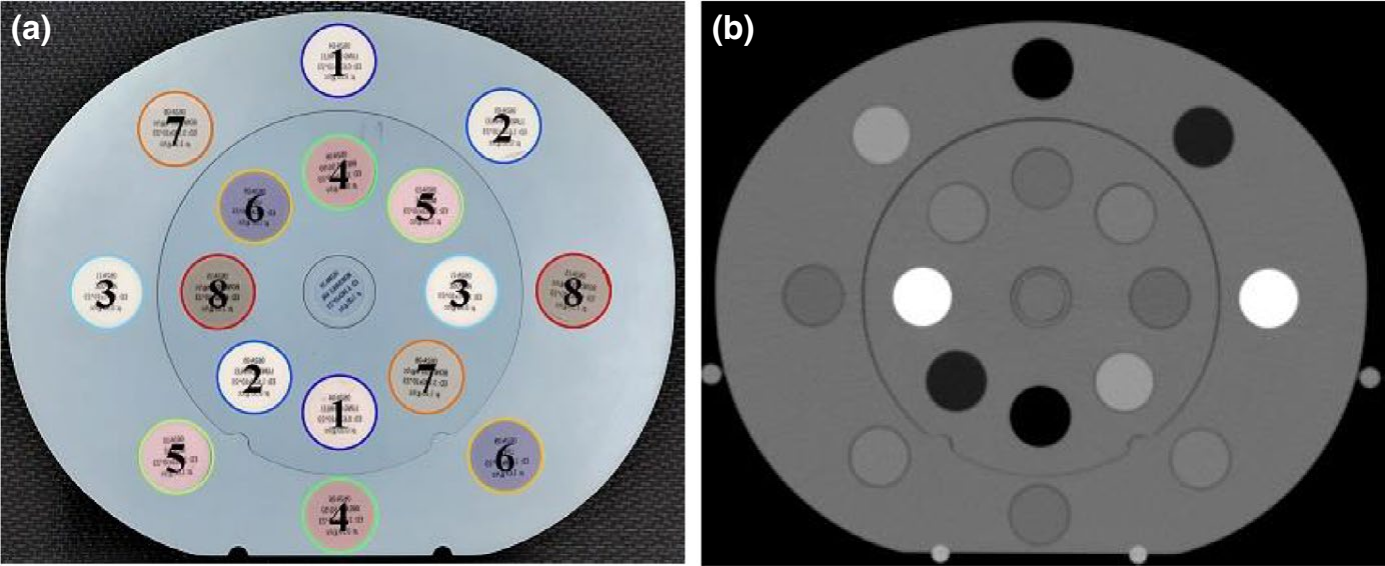
(a) Electron density phantom photograph and (b) planning computed tomography image: 1, lung (inhale); 2, lung (exhale); 3, adipose; 4, breast; 5, muscle; 6, liver; 7, trabecular bone; and 8, solid dense bone

The electron density (ED) phantom (CIRS Model 062MA, CIRS Tissue Simulation Technology, Norfolk, USA) was used to measure the 3D CT value; it had a width of 330 mm, height of 270 mm, and length of 250 mm. The tissue‐equivalent‐substance plugs (ED plugs) inserted into the phantom were 3 cm in diameter and 5 cm in length. Eight tissue equivalents were placed concentrically on the outer and inner head inserts (Figure 1). The ED plugs were placed as both inner and outer plugs, which is suitable for evaluating CT value characteristics due to cupping artifacts and beam hardening effect. Table 1 shows the tissue equivalents, physical densities, and relative ED (RED) in the product guide.

**TABLE 1:** Physical density of each ED phantom insert material obtained from phantom's specifications

Material description	Physical density (g/cc)	Relative ED
Lung (inhale)	0.205	0.200
Lung (exhale)	0.507	0.496
Adipose	0.96	0.949
Breast	0.99	0.976
Muscle	1.06	1.043
Liver	1.07	1.052
Trabecular bone	1.16	1.117
Solid dense bone	1.53	1.456

Abbreviation: ED, electron density.

### 2.2 Image acquisition

Three‐dimensional CT values from a CBCT were measured under three different imaging conditions, with characteristics as presented in Table 2. Depending on the size of the field of view (FOV), the conditions were the following: head and neck (S‐scan), chest (M‐scan), and pelvis (L‐scan). The S‐scan was a half‐scan in which images were acquired by rotating the gantry by 200° and captured by inserting a non‐bowtie F0 cassette. Therefore, the S‐scan was acquired only with the inner head insert. Conversely, the M‐scan and L‐scan used an F1 bowtie filter to perform a full scan that acquired images by rotating the gantry by 360°. The size of the FOV was modified by placing the flat‐panel detector at an offset with respect to the X‐ray beam center. For this reason, the S‐scan with the smallest FOV was positioned with the flat‐panel detector at the center of the X‐ray beam center.

**TABLE 2:** Clinical exposure protocol

Scan mode	S‐scan	M‐scan	L‐scan
X‐ray voltage (kV)	100	120	120
X‐ray current per projection (mA/frame)	10	40	64
Exposure time (ms/frame)	10	40	40
Number of projections	183	660	660
Total (mAs)	36.6	1056.0	1689.6
Collimator	Small	Medium	Large
Filter	F0	F1	F1
FOV (mm)	270	410	500

Abbreviation: FOV, field of view.

The M‐scan had an offset of 115 mm and an FOV of 410 mm, whereas the L‐scan had an offset of 190 mm and a maximum FOV of 500 mm. The longitudinal maximum distance of each imaging method was 276.7 mm, and it was possible to acquire 3D volumetric data for the ED phantom. However, because the ED plugs were only 5‐cm long in the longitudinal direction, the same scan was performed eight times while moving the plug in the longitudinal direction by approximately 3 cm per repetition. Using these images, a simulation was performed so that the ED plugs were present in all the sections. The ED phantom was similarly entirely imaged using pCT under imaging conditions as per the general treatment plan CT using the following system specifications: tube voltage 120 kV, tube current 150 mA, X‐ray tube rotation speed 0.75 s/rotation, pitch factor 0.688, collimation 1.25 mm × 16 detector array, set slice thickness 5 mm, image reconstruction filter function FC13 (standard abdominal kernel), and FOV 390 mm.

### 2.3 CT value error analysis

First, the CT value was measured in the isocenter axial slice from the ED phantom using pCT and CBCT imaging data. For this, a region of interest with a diameter of 16 mm was set for all ED plugs. The scan was repeated three times, and the CT values for each scan were averaged. As shown in Table 1, each ED plug has a corresponding RED. Therefore, a RED curve was generated from the measured CT values and compared for pCT and the CBCT. M‐scanning and L‐scanning were used to compare the CT values of the inner and outer ED plugs. Next, the ED phantom was imaged under the conditions shown in Table 2 to evaluate the 3D CT value error in the CBCT. Subtraction images from pCT and the CBCT were generated using ImageJ (National Institutes of Health) software for analysis of CT value errors. The CT value error profiles of the horizontal and vertical lines were calculated and evaluated from the subtraction image of the isocenter axial slice. Similarly, the CT value error profile in the longitudinal direction was measured. Longitudinal profiles were measured for ED plugs representing a lung (inhale), breast, and solid dense bone. Lung and breast materials have similar physical densities for CT value correction, and solid dense bone has the highest physical density of all the materials. The L‐scan and M‐scan measurements were obtained from the inner and outer plugs.

### 2.4 Evaluation of dose calculation

The evaluation of pCT and CBCT doses was performed using Monaco. The CBCT dose was calculated using images to evaluate the effect of the CT value error of the CBCT on dose calculation. The respective images of pCT and the CBCT were transferred to the Monaco version 5.11.03 treatment planning system (Elekta Ltd). The treatment plan was delivered to the phantom with an irradiation field of 40 cm ×40 cm using single field, two parallel opposed fields, 4‐field box, and single‐arc techniques. Dose distributions were calculated using a Monte Carlo algorithm, XVMC, available in Monaco version 5.11.03, with a grid size of 3 mm. The prescribed dose was 50 Gy for the phantom center, and a simple treatment plan was determined without any detailed optimization. All dose calculation was performed using ED values determined from the CT to ED calibration curves of pCT. To evaluate the dose distributions in the CBCT images, the treatment plan created on the pCT was imported to the CBCT images with the same parameters. Dose errors were calculated by subtracting the S‐scan, M‐scan, and L‐scan dose distributions from the pCT dose distribution using the Monaco treatment planning system. The average dose error of the ED plugs was measured using subtraction images. The measurement was performed by setting a region of interest with a diameter of 16 mm for all ED plugs in the isocenter axial slice in the subtraction image. To perform a detailed evaluation of the dose distribution surrounding the ED plug, the dose error profiles of the horizontal and vertical lines were calculated from the subtraction images of the isocenter axial slices.

## 3 RESULTS

### 3.1 CT value to RED curves

**FIGURE 2:**
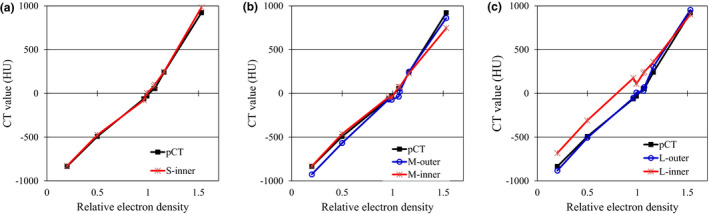
Comparison of computed tomography (CT) to the relative electron density curves in planning computed tomography (pCT) and cone beam computed tomography: (a) S‐scan, (b) M‐scan, and (c) L‐scan

Figure 2 shows a comparison of the curves of the CT value and the RED of the material. On the basis of the CT values from pCT, the S‐scan shown in Figure 2a showed small CT value errors (S‐inner) for all materials. The highest error was 59.8 ± 22.4 HU (mean ± standard deviation) in solid dense bone. In the M‐scan shown in Figure 2b. M‐inner showed an error of 176.0 ± 7.3 HU at high RED, and M‐outer showed an error of 90.4 ± 4.8 HU at low RED. In the L‐scan shown in Figure 2c, the outer phantom CT values (L‐outer) agreed well. In the inner phantom, a large CT value error of approximately 179.6 ± 34.9 HU was obtained, except in the bone material.

### 3.2 CT value error distribution

**FIGURE 3:**
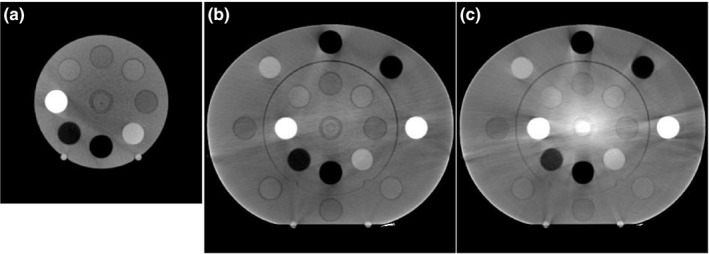
Cone beam computed tomography images acquired using each protocol: (a) S‐scan, (b) M‐scan, and (c) L‐scan

Figure 3 shows CBCT images at the isocenter where the ED phantom was imaged under each condition. The L‐scan shown in Figure 3 is nonuniform due to the high CT value at the isocenter in the axial slice. The S‐scan and M‐scan were more uniform than the L‐scan. In all CBCT images, significant streak artifacts extending linearly from bone and lung materials were apparent. The L‐scan increased the CT value at the center of the phantom by about 800 HU due to in‐plane cupping artifacts (Figure 3c).

**FIGURE 4:**
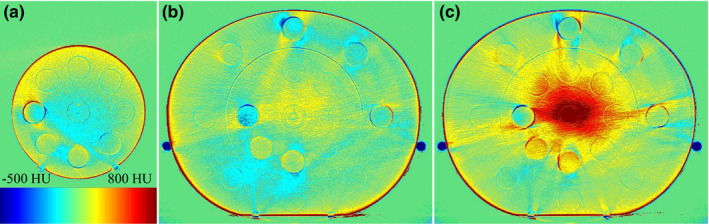
Computed tomography value errors in subtraction images acquired for each protocol: (a) S‐scan, (b) M‐scan, and (c) L‐scan

Figure 4 shows pCT and CBCT subtraction images. With pCT, uniform images were obtained for all slices (Figure 1b). Therefore, in the subtraction image, nonuniform areas and artifacts resulted in CT value errors. The L‐scan (Figure 4c) CT value showed a significant error at the isocenter. Therefore, all the materials measured had positive inner CT value errors except for solid dense bone. The S‐scan had a slightly lower CT value at the isocenter than off‐center.

### 3.3 CT value error profile

**FIGURE 5:**
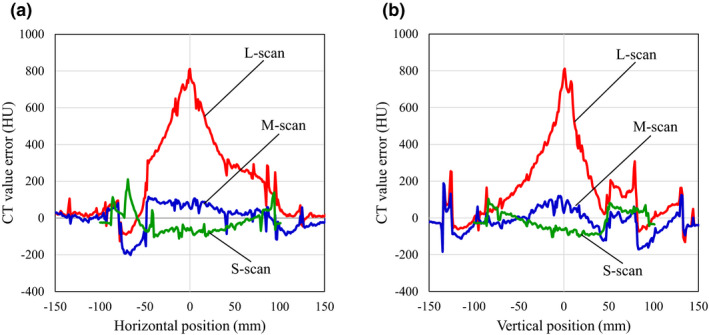
Comparison of computed tomography (CT) value error profiles by scan mode: (a) horizontal profiles and (b) vertical profiles

**FIGURE 6:**
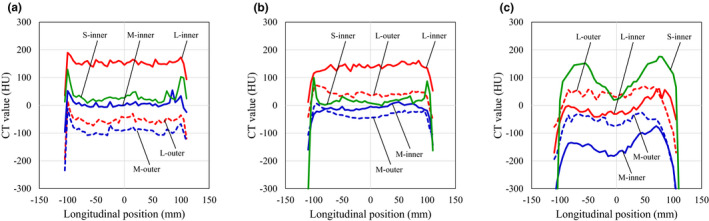
Comparison of longitudinal computed tomography (CT) value error profiles by scan mode: (a) lung (inhale), (b) breast, and (c) solid dense bone

Figures 5 and 6 show the CT value error profiles in the subtraction image (Figure 4). The profiles are shown in the horizontal and vertical directions across the center of the phantoms (Figure 5). The L‐scan had a maximum CT value error of 813.7 HU at the isocenter compared with the off‐center in the axial slice. In addition, the M‐scan had an inner CT value error of −195.4 ± 4.9 HU in the solid dense bone material (Figure 5a), and the lung (inhale) material on the L‐scan had a CT value error of 226.5 ± 72.2 HU (Figure 5b). Otherwise, the CT value error was approximately ±100 HU.

Figure 6 shows the CT value error present along the longitudinal direction. Profiles were measured for (a) lung (inhale), (b) breast, and (c) solid dense bone materials. The CT values measured for material inside the phantom via the M‐scan and L‐scan were denoted M‐inner and L‐inner, respectively, whereas the external material's measurements are denoted M‐outer and L‐outer, respectively. Similar to the results in Figure 4c, the L‐scan tended to have higher inner CT values in the lung (inhale) material (Figure 6a) and breast material (Figure 6b). Lung (inhale) was approximately 153.7 ± 7.8 HU higher than pCT, and breast was 140.6 ± 11.1 HU higher. The lung (inhale) and breast materials were almost uniform in the longitudinal direction under all imaging conditions, but a nonuniform profile was present in the solid dense bone material (Figure 6c). In particular, S‐inner was 156.4 HU higher than at the center, and M‐inner was 108.8 HU higher off‐center than at the center.

### 3.4 Dose calculation error

**FIGURE 7:**
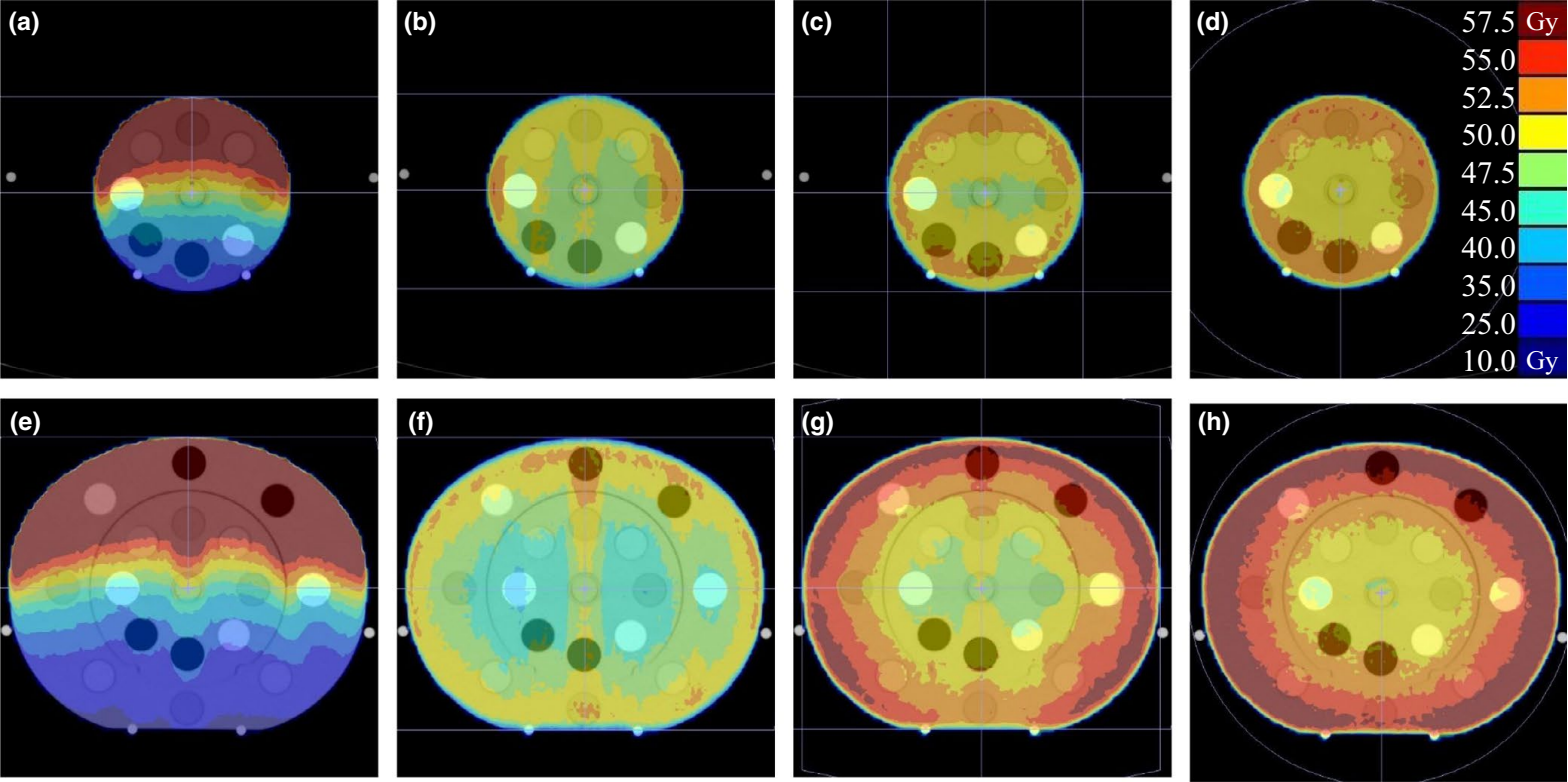
Dose distribution of planning computed tomography planned using each irradiation technique. The upper row shows the cases of the (a) single field, (b) two parallel opposed fields, (c) 4‐field box, and (d) single arc for the inner head insert. The lower row shows the cases of the (e) single field, (f) two parallel opposed fields, (g) 4‐field box, and (h) single arc for the electron density phantom

Figure 7 shows the dose distribution of pCT planned using each irradiation technique. The upper row figures show the cases of the (a) single field, (b) two parallel opposed fields, (c) 4‐field box, and (d) single arc for the inner head insert. The lower row figures show the cases of the (e) single field, (f) two parallel opposed fields, (g) 4‐field box, and (h) single arc for the ED phantom.

**FIGURE 8:**
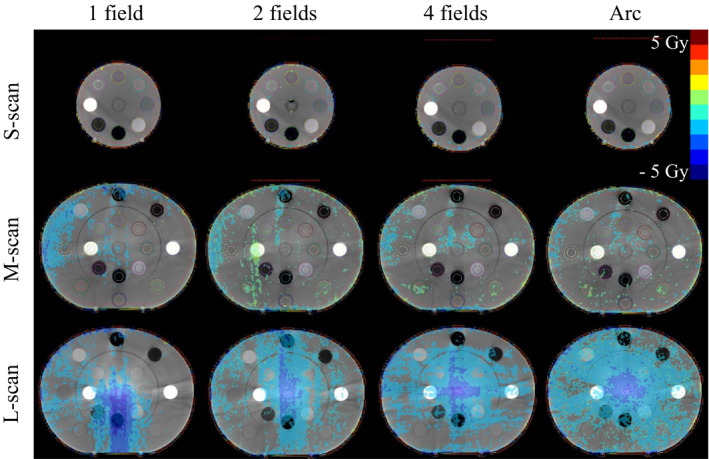
Subtraction image of the planning computed tomography and cone beam computed tomography dose distributions. S‐scans, M‐scans, and L‐scans are shown in the upper, middle, and lower rows, respectively. The irradiation techniques are (from left to right) single field, two parallel opposed fields, 4‐field box, and single arc

Figure 8 shows the subtraction images of pCT and CBCT doses for the isocenter, where the CBCT dose is evaluated for S‐scan, M‐scan, and L‐scan with single field, two parallel opposed fields, 4‐field box, and single‐arc treatment planning dose distributions. The dose error for ±100 HU difference in CT values for the S‐scan and M‐scan was within ±1 Gy. In this study, we set the prescription dose to 50 Gy; thus, the dose error was within ±2%. The solid bone inner plug for the M‐scan had a CT value error of approximately 200 HU, but the dose error was within ±2%. The center of the L‐scan had a CT error of approximately 800 HU and a dose error of approximately 6%. The dose error of the L‐scan occurred in the beam path, and the maximum error occurred at the center of the phantom in the case of both the 4‐field box and single‐arc techniques.

**FIGURE 9:**
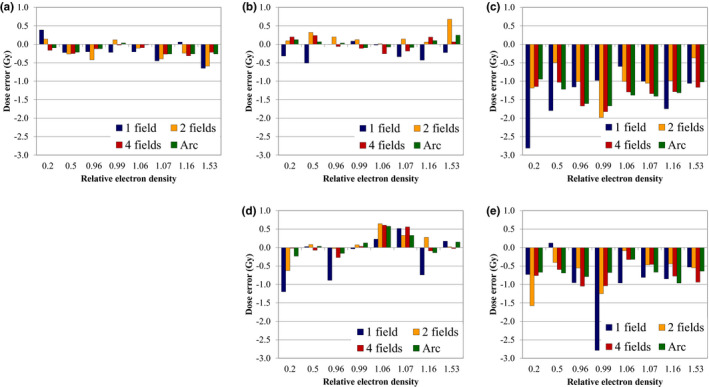
Average dose error of the ED plug in subtraction images under each imaging condition: (a) S‐scan, (b) inner plug of M‐scan, (c) inner plug of L‐scan, (d) outer plug of M‐scan, and (e) outer plug of L‐scan.

Figure 9 shows the average dose at each ED plug calculated from the subtraction images obtained in Figure 8. As shown in Figure 9a,b,d, the dose errors for the S‐scan and M‐scan were within 1 Gy. Most inner plugs of the L‐scan shown in Figure 9c showed a dose error of >1 Gy. The outer plug of the L‐scan shown in Figure 9e exhibited a dose error of 2.8 Gy for the mammary plug in one field; in the two fields, the lung and mammary plugs showed dose errors exceeding 1 Gy; however, the other plugs showed dose errors that were almost within 1 Gy.

## 4 DISCUSSION

In this paper, we evaluated the CBCT HU error in three dimensions by changing the scan mode of the CBCT. Moreover, the CBCT dose error associated with the CBCT HU error was evaluated by changing the irradiation techniques. CBCT HU errors (Figure 4) directly reflected the effect of CBCT‐specific artifacts (Figure 3) in the axial plane homogeneous pCT image (Figure 1b). The CBCT HU error due to axial plane nonuniformity was approximately ±100 HU for the S‐scan and M‐scan (Figure 5). However, the L‐scan had a CT value error of approximately +800 HU at the isocenter compared with off‐center in the axial plane. This is because it used the same bowtie filter as the M‐scan, whereby proper beam hardening correction was not performed, and cupping artifacts are hence assumed to have occurred. Therefore, the CBCT HU for inner materials via the L‐scan showed a tendency toward higher values, except for the bone (Figure 6). Further, the CBCT HU in the longitudinal direction was also uniform except for the bone. The anode and cathode of the X‐ray tube were vertical to the longitudinal plane, and the heel effect^7^ thus affected the X‐ray intensity of the axial plane; conversely, there were few longitudinal directions. Because the CT value changes depending on photon energy,^25^ it is thought that the effect on the CT value's error is small in the longitudinal direction, which is less affected by the heel effect. However, the bone is susceptible to CT values affected by photon energy.^27^ Therefore, it is considered that the CBCT HU error was larger at the off‐center positions than at the isocenter due to beam hardening in the S‐scan without a bowtie filter (Figure 6c). Richter et al reported a CT dose error of up to 14.5% due to incompatible CBCT scan parameters,^25^ whereas our evaluation showed a maximum L‐scan CBCT dose error of 6%. This reduction was considered to be due to adding the CT value correction in the device used, which reduced the dose error.

S‐scans and M‐scans with a CBCT HU error of approximately ±100 HU had a CBCT dose error of approximately 2%. The L‐scan, with a CBCT HU error of approximately 800 HU, had a CBCT dose error of approximately 6%. Srinivasan et al reported a 3% dose error due to differences in the correction phantoms,^5^ suggesting that the S‐scan and M‐scan were properly calibrated. Dose errors should be <3%–5% for tumor control in clinical practice.^28^, ^29^ The cause of the cupping artifacts in the L‐scan is thought to be a mismatch between scan mode and phantom size. Moreover, the M‐scan and L‐scan should have been properly corrected for beam hardening using a different bowtie filter, but the Synergy XVI system uses the same filter for both scans, which may have caused the CBCT HU error. Richter et al evaluated the CBCT HU error for the consequent CBCT dose error only for the in‐axis plane.^25^ However, we have performed a detailed evaluation for the longitudinal direction as well. The bone is prone to CBCT HU errors in the longitudinal direction, and it is a new finding that the change was particularly noticeable in the S‐scan without a bowtie filter. Furthermore, Abe et al measured the CBCT's values in the longitudinal direction using patient images.^26^ Therefore, determining whether the CBCT HU error is caused by a patient's size or by scan parameters is difficult. In our study, we used a phantom and found that the scan mode affected the longitudinal direction.

We evaluated the CBCT dose error associated with CBCT HU error by changing the irradiation techniques. Li et al have evaluated the effect of CT value errors caused by artifacts on the dose distribution in a single field irradiation technique.^30^ However, we evaluated the CBCT dose error with several irradiation techniques. As shown in Figure 2c, the CT value error of L‐outer was within ±100 HU; however, the dose error was more than 1 Gy in the single‐field and two‐field cases. As a result, it is clear that the CBCT dose error increases from the starting point of the CBCT HU error to the peak (Figure 8). For a single field of the L‐scan, the CBCT dose error is located along the beam path beyond the peak of the CBCT HU error, whereas the dose errors of the 4‐field box and single arc converge to the isocenter.

The clinical treatment plan positions the target at the isocenter. Therefore, careful attention should be paid to cupping artifacts. This is because the peak of the CBCT dose error occurs at the target isocenter. In particular, cupping artifacts are significant in L‐scans, for which CT value correction is not properly performed. Therefore, if used for ART without proper CT value correction, it may affect not only the target dose but also the surrounding normal tissue dose.^31^

A limitation of this study is that we did not examine whether the bowtie filter, tube voltage, FOV, phantom size, or tube direction was the cause of the error in the CT values. Nevertheless, this is an important report that uses a clinical machine to evaluate in detail the 3D CBCT HU error and the associated CBCT dose error with the addition of overall effects.

## 5 CONCLUSION

We evaluated the HU CBCT error using the CBCT mounted on the linac in various scan modes and found significant errors in HU values, comparable with those in a pCT. The largest CBCT HU error was 800 HU in the L‐scan, which is considered to be a cupping artifact due to phantom size mismatch. The CBCT HU of the bone, which is affected by photon energy, increased the error in the longitudinal direction, and S‐inner was up to 156.4 HU higher in CT value at the longitudinal off‐center than the isocenter. The reason may be that S‐inner has no bowtie filter. Moreover, the CBCT dose error associated with the CBCT HU error was evaluated by changing the irradiation techniques. For a single field of the L‐scan, the 6% CBCT HU error occurred beyond the peak, and the CBCT dose error is located along the beam path beyond the peak, whereas the CBCT dose errors for the 4‐field box and single arc converge to the isocenter.

We have demonstrated the effect of changing the scan mode of the Elekta XVI CBCT on the accuracy of the dose calculation owing to CT value errors in three dimensions.

## CONFLICT OF INTEREST

The authors declare no conflict of interest.

## AUTHOR CONTRIBUTIONS

All authors discussed the results and contributed to the final manuscript. T. H., T. S., F. H., Y. N., H. K., O. T., and M. M. helped supervise the project. K. O. and S. N. carried out the experiment.
